# Climate, health, and living condition crises in the expanding informal settlements and slums of South-West Nigeria: a case report of Ogun and Oyo states

**DOI:** 10.7189/jogh.15.03031

**Published:** 2025-06-27

**Authors:** Isaac Olufadewa, Olumide Abiodun, Ruth Oladele, Okechukwu I Eze, Queen Adeyemo, Joshua Omale, Lawrence Nnyanzi, Akinyimika Sowunmi, Boni Maxime Ale, Akindele O Adebiyi, Davies Adeloye

**Affiliations:** 1 Slum and Rural Health Initiative, Ibadan, Nigeria; 2 College of Medicine, University of Ibadan, Ibadan, Nigeria; 3 Department of Community Medicine, Babcock University, Ilishan-Remo, Nigeria; 4 Teesside University International Business School, Middlesbrough, UK; 5 Centre for Public Health Research, School of Health and Life Sciences, Teesside University, Middlesbrough, UK; 6 Holo Global Health Research Institute, Nairobi, Kenya; 7 Health Data Acumen, Nairobi, Kenya

## Abstract

The rapid expansion of informal settlements and slums in southwestern Nigeria, particularly along the Lagos-Ogun-Oyo corridor, underscores the critical challenges posed by rapid urbanisation and population growth in this major economic hub of West Africa. This corridor, home to key infrastructure, industries, and economic activities, has seen a significant rise in unplanned communities due to its economic opportunities. In particular, Ogun and Oyo states, both sharing borders with Lagos, have become hotspots of these communities, characterised by inadequate housing, poor sanitation, unsafe drinking water, and heightened vulnerability to climate-related hazards. In this viewpoint, we examine the health and environmental challenges faced by residents in these informal settlements, including respiratory illnesses, waterborne diseases, and climate-related health risks exacerbated by poor air quality and extreme heat. Despite various research efforts, policy reforms, and programmatic interventions, challenges such as limited funding, inadequate enforcement, and a lack of coordination among stakeholders persist. We propose a holistic, multi-sectoral approach that could improve living standards and health outcomes through community empowerment, participatory urban planning, microfinance initiatives, and climate resilience programmes. We note that this requires collaborative efforts from government, non-governmental organisations, and local residents to create sustainable and resilient urban environments.

Nigeria, the most populous country in Africa with over 223 million inhabitants, is grappling with significant challenges arising from rapid urbanisation and population growth [1]. In 2012, the United Nations projected that its population would double by 2050, positioning the country as the third most populous globally [2,3]. This rapid demographic expansion, coupled with insufficient infrastructure development, has led to a rise in informal settlements and slums in many Nigerian urban cities. These areas are characterised by inadequate housing and heightened health risks, disproportionately affecting vulnerable populations such as women, children, and the elderly.

Ogun and Oyo states, located in southwestern Nigeria, had a combined population of 14.4 million in 2022. [1]. Both have become emerging hubs for informal settlers [4], primarily due to their proximity to Lagos State, which has an estimated population nearing 25 million [1]. The Lagos-Ogun-Oyo corridor encompasses critical economic infrastructure in Nigeria, including many manufacturing companies, multinationals, banks, large markets, the Lagos-Ibadan Expressway (the busiest highway in West Africa), the Apapa Port, and Murtala Muhammed International Airport. The Lagos-Ibadan Expressway also serves as a major connection route between Nigeria’s northern, eastern, and southern parts. Consequently, economic opportunities along this corridor contribute to ongoing population growth and continued rural-urban migration, which subsequently trickles down to the rise in informal settlements and slums (**Figure 1**).

**Figure 1:**
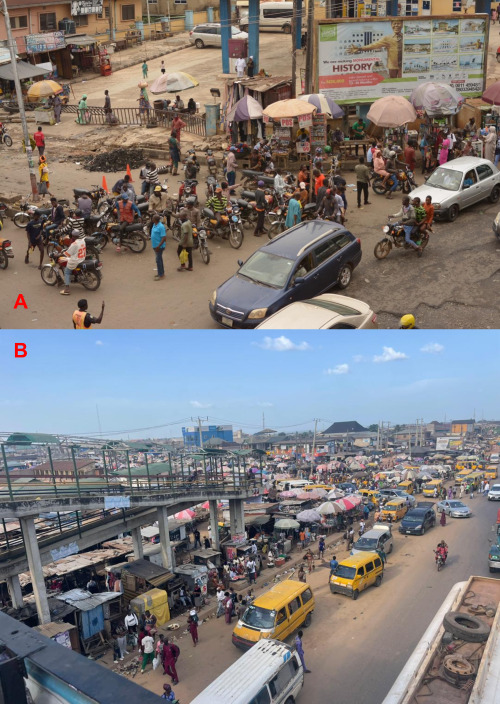
A hub of transport workers in slums. **Panel A.** Motorcycles (Ibadan, Oyo). **Panel B.** Cars and Buses (Mowe-Ibafo, Ogun).

Additionally, the escalating cost of living in Lagos has driven many residents to seek more affordable housing in adjacent satellite communities while continuing to leverage economic opportunities in Lagos itself. The Mowe-Ibafo corridor along the Lagos-Ibadan Expressway serves as a prominent example of this urban shift, now characterised by expanding informal settlements and slums. These shifts have contributed to the proportion of the population living in cities in Nigeria increasing from 9.4% in 1950 to 52% in 2020, while the percentage of people living in slums and informal settlements in Nigeria is projected to rise from 48% in 2019 to 52% by 2024, a figure that may be underestimated due to the lack of comprehensive and representative data [5].

Key issues plaguing these informal settlements include substandard housing, inadequate sanitation, unsafe drinking water, and poor waste disposal practices. These conditions are known to elevate the disease burden, contributing to public health crises [6]. Additionally, climate risks such as extreme heat and air pollution exacerbate the already substandard living conditions, further deteriorating the overall health status of residents.

In this viewpoint, we examined the health and environmental challenges present in the informal settlements of Ogun and Oyo states. We further highlighted the gaps in research, policy, and programmatic interventions, and proposed recommendations to improve living standards and health outcomes for residents.

## EXPLORING CLIMATE AND HEALTH RISKS IN THE INFORMAL SETTLEMENTS OF OGUN AND OYO STATES

Informal settlements in Ogun and Oyo states, much like others across Africa, are marked by a confluence of environmental, social, and infrastructural deficits that significantly elevate the risk of adverse health outcomes [7]. Inadequate housing, poor sanitation, and limited access to clean water contribute to a high burden of disease, including respiratory illnesses, waterborne diseases, and conditions exacerbated by climate risks such as poor air quality and extreme heat [7,8].

### Respiratory illnesses

A common health issue among residents of informal settlements is the high incidence of respiratory illnesses, as reported in a previous meta-analysis [9]. These conditions are driven by several factors, including poor indoor air quality, overcrowded living conditions, and proximity to pollution sources. Inadequate ventilation and the widespread use of biomass fuels, such as wood and charcoal for cooking, contribute to high levels of household air pollution [10], which is particularly pronounced in slums [11,12]. In Ogun and Oyo states, similar patterns are likely observed, given the reliance on traditional cooking methods and the proximity of settlements to industrial zones, highways, and congested urban areas, all of which exacerbate air pollution [7]. Additionally, poor outdoor air quality, driven by vehicle emissions from major highways like the Lagos-Ibadan Expressway and industrial activities in the Lagos-Ogun-Oyo corridor, may further worsen respiratory health.

### Waterborne diseases

Access to clean water remains a critical challenge in informal settlements. Residents often rely on unsafe water sources such as unprotected wells, rivers, and rainwater, which are prone to contamination by human and animal waste due to poor sanitation and inadequate waste disposal systems.[13] As a result, waterborne diseases such as cholera, typhoid, and diarrheal diseases are prevalent in these communities. A study conducted in the Kibera slum in Nairobi, Kenya, found that diarrhoeal diseases were one of the leading causes of morbidity and mortality, particularly among children under five [14]. Similarly, from our observations in the informal settlements of Ogun and Oyo states, limited access to clean water and inadequate sanitation systems create a conducive environment for the spread of waterborne pathogens, potentiating the reality of frequent outbreaks of these diseases [7,8]. Flooding, often associated with poor drainage systems and climate change-induced extreme weather, exacerbates the issue through contamination of surface and groundwater sources. This is a common scenario during the rainy season, which leads to spikes in cholera and other waterborne infections [15].

### Climate risks: poor air quality and adverse heat

Climate change presents additional health challenges in informal settlements, particularly regarding air quality and temperature extremes. Urban heat islands, exacerbated by dense, unplanned settlements with limited green spaces, also significantly increase heat-related illnesses [16]. In many settings, their effects can raise local ambient temperatures by 2–4°C above surrounding areas, with peak daytime ambient temperatures in West African cities sometimes exceeding 40–45°C [17]. This issue is particularly acute in Ogun and Oyo states, where informal settlements often lack proper ventilation, cooling systems, or shade, exposing residents to indoor temperatures that can reach 35–40°C during hot seasons [18]. The impacts of extreme heat on informal settlers are well-reported [10,12], with heatwave periods linked to 15–25% rises in heat-related hospital admissions and notable increases in heat stroke and dehydration cases; pre-existing conditions such as hypertension and cardiovascular diseases see exacerbations of 10–20% in incidence or severity during peak heat [18,19]. The interaction between poor air quality and rising temperatures also worsens respiratory conditions. Studies in southwestern Nigeria have shown that higher temperatures can increase the concentration of ground-level ozone by about 10–20% on hot days and elevate particulate matter levels by 5–10 μg/m^3^ above baseline [18,20,21]. In informal settlements, where residents are already exposed to indoor and outdoor air pollution, these climate-related increases can translate to measurable upticks in clinic visits for asthma exacerbations or acute respiratory infections [7,18].

## EXPLORING RESEARCH, POLICIES, AND PROGRAMMES

### Research

Research is vital for understanding the complexities of informal settlements and guiding the development of effective policies and programmes [22]. The Nigerian Institute of Social and Economic Research has conducted valuable studies assessing the living conditions, housing demands, and socioeconomic factors influencing informal settlements in Oyo and Ogun states [23]. These studies provide crucial insights into the challenges faced by residents, including inadequate infrastructure, overcrowding, and health risks. Additionally, the World Bank has focussed on urbanisation trends and housing challenges across Nigeria, offering comprehensive data that can inform national and regional policy decisions [24]. Such research is instrumental in identifying areas that require intervention and informing policy frameworks that address the unique needs of these communities.

Some local governments have also undertaken research initiatives, focussing on data collection specific to informal settlements [25]. These efforts help pinpoint the specific challenges of each settlement, such as access to clean water, sanitation, health care, and housing. However, these local initiatives often face resource constraints that limit the scope and depth of the data collected.

Despite the significance of research in shaping policy, several challenges hinder its effectiveness. A major issue is the availability and quality of data, which we already noted earlier [26]. Collecting reliable data in informal settlements can be difficult due to logistical constraints, including the transient nature of residents and their reluctance to participate in surveys, which can result in incomplete or skewed data. This limitation often leads to misinformed policies that do not adequately address the reality on the ground. Furthermore, there is a persistent gap between research findings and their application in policy and practice. Even when research produces valuable insights, these recommendations may not be effectively communicated to policymakers or may be deprioritised, resulting in a disconnect between research and action. This challenge is compounded by the project-based nature of many research initiatives, which are often short-term and lack sustainability. Consequently, the momentum for ongoing data collection, analysis, and policy adjustment is lost, hindering long-term improvements in the health and living conditions of informal settlements.

### Policies

The Nigerian government has introduced several policies aimed at improving housing and living conditions in informal settlements. The National Housing Policy emphasises the importance of providing affordable housing and promoting urban renewal across Nigeria [27]. It seeks to address the housing deficit by encouraging public-private partnerships and slum upgrading initiatives. In Oyo State, the Oyo State Housing Policy outlines strategies for housing development and slum upgrading, with a focus on enhancing living conditions for residents in informal settlements [28]. Similarly, the Urban Development Law of Ogun State provides a framework for urban planning, regulating land use, and addressing the challenges posed by informal settlements [29].

Despite these policy frameworks, their implementation faces several critical challenges. One of the major obstacles is the inadequate legislative framework, particularly in the enforcement of housing standards. In addition, the lack of effective enforcement mechanisms allows informal settlements to proliferate without adequate oversight or regulation. Without strong legal structures, efforts to improve housing quality and ensure compliance with urban planning regulations often fail. Moreover, there is a frequent lack of political will to prioritise housing and informal settlement issues. Thus, even when policies are in place, the commitment from political leaders to drive these policies forward is often inconsistent, with other political or economic issues taking precedence. This lack of sustained attention undermines the effectiveness of housing policies and leads to sporadic or incomplete interventions in informal settlements.

Another significant challenge is the lack of coordination among key stakeholders, including government agencies, non-governmental organisations (NGOs), and community-based organisations. Fragmented approaches, coupled with insufficient collaboration, frequently lead to duplicated efforts or, conversely, significant gaps in service delivery. The absence of a cohesive strategy and clear communication channels between these groups further hampers the successful implementation of policies aimed at upgrading informal settlements.

### Programmes

Several targeted programmes have been implemented in Oyo and Ogun States to address the challenges faced by residents of informal settlements. One notable initiative is the Oyo State Urban Renewal Programme, which seeks to improve infrastructure, sanitation, and housing conditions in slum areas through the rehabilitation and construction of new facilities [30]. It aims to revitalise urban spaces by addressing the infrastructure deficits that contribute to poor living conditions in informal settlements.

The African Development Foundation has also launched projects focussed on enhancing access to clean water and sanitation facilities, which are critical in reducing health risks associated with poor hygiene and living conditions in informal settlements [31]. This initiative has helped to improve public health by targeting one of the core issues: the availability of safe drinking water and proper sanitation.

NGOs also play a significant role in improving living conditions. For example, the Oyo State Community Development Agency has initiated several community-driven programmes that include vocational training to economically empower residents and improve their overall quality of life [32]. These programmes help equip residents with skills that enable them to find employment or start small businesses, addressing the economic challenges faced by many informal settlers.

The Slum and Rural Health Initiative (SRHIN) has undertaken multiple projects aimed at addressing health and social issues within informal settlements. Programmes like ‘*Beta Mama Pikin*’ (translated as ‘Better Mother and Child’) focus on maternal and child health, while ‘Food in Slums’ works to improve nutrition for vulnerable populations [33]. Other initiatives, such as Sports for Street Children and Mental Health Education, emphasise mental health awareness and employability, especially among the youth. By working with local, state, and national stakeholders, SRHIN strives to holistically improve the lives of slum residents through these multifaceted interventions.

While these programmes have noble intentions and have made meaningful contributions, several challenges hinder their overall effectiveness. One major issue is that many programmes only address small components of the larger problem, often due to resource constraints. Implementing a comprehensive, multi-component intervention requires significant financial and logistical investment, which many initiatives lack. For instance, the Oyo State Urban Renewal Programme has faced budgetary constraints, limiting its capacity to fully upgrade infrastructure and improve living conditions in a sustainable manner [34].

Moreover, many interventions fail to address the specific needs of the communities they aim to serve without actively involving residents in the planning and decision-making processes. Low awareness of available programmes, coupled with insufficient communication between residents and programme organisers, leads to poor participation and reduces the overall impact of these initiatives. The Oyo State Urban Renewal Programme, while ambitious, has been criticised for its slow implementation and limited scope [34]. Residents often report being unaware of the available support or feel disconnected from the initiatives designed to improve their living conditions [35].

Additionally, many programmes tend to focus heavily on physical infrastructure improvements, such as roads and housing, without incorporating the necessary social and economic components, such as health services, education, and employment opportunities. This approach often results in limited, short-lived impacts on residents’ quality of life. Sustainable improvements require an integrated approach that addresses both physical and social needs simultaneously.

## WAY FORWARD

### Capacity building and community empowerment

Empowering residents through capacity-building initiatives is essential for improving living conditions in informal settlements. Programmes that focus on skills development, financial literacy, and entrepreneurship can provide individuals with the tools to create sustainable livelihoods. For instance, vocational training in trades such as carpentry, plumbing, tailoring, and electrical work would enable residents to secure employment or start small businesses within their communities. These initiatives could also integrate ‘Waste to Wealth’ programmes, where waste management is turned into income-generating activities. Such interventions could simultaneously improve living conditions and increase the earning potential of families. Collaboration between the government, private sector, and NGOs is critical to scaling these initiatives and ensuring their sustainability [36].

Another important aspect of community empowerment is access to financial support and advisory services for women and young people. Programmes that provide microgrants or seed funding for small businesses, particularly those led by women, can have significant social and economic ripple effects, helping to address poverty, unemployment, and the overall well-being of households.

### Participatory urban planning

To create lasting and effective solutions for informal settlements, residents must be actively involved in the urban planning process. Participatory urban planning is a bottom-up approach that encourages collaboration between community members, local government, NGOs, and other stakeholders. It ensures that residents’ needs and aspirations are reflected in urban development strategies. When residents have a say in decisions regarding housing, infrastructure, and service delivery, they are more likely to support and sustain these improvements [37].

Participatory urban planning also fosters a sense of ownership and accountability among residents, which can reduce the resistance to change often observed in top-down interventions. For example, involving residents in discussions around sanitation, waste management, and housing upgrades could lead to more contextually appropriate solutions, ensuring a higher rate of success in project implementation [38]. This collaborative approach also allows for the incorporation of local knowledge and experience, which can help address specific environmental and socioeconomic challenges in informal settlements.

### Microfinance and cooperative societies

Access to financial resources is a significant barrier for many informal settlement residents looking to improve their living conditions [39]. National survey data show that a large share of households in southwestern Nigeria, particularly in urban low-income and informal areas, have monthly expenditures below the poverty threshold and rely predominantly on informal, irregular earnings [40].

Establishing microfinance institutions and cooperative societies tailored to the needs of these communities directly addresses this specific deficit in access to affordable credit. Microfinance services would provide low-interest or flexible loans to individuals and groups, enabling them to invest in housing improvements, sanitation upgrades, and small-scale income-generating activities. Cooperative societies could serve as a collective platform for residents to pool savings and jointly invest in infrastructure or business ventures, matching the observed preference and practice of communal risk-sharing in informal settlements.

Microfinance programmes have been successful in many areas in Nigeria. For example, documented welfare improvements among small-scale entrepreneurs in Oyo State and rural households in southwestern Nigeria suggest these models can be adapted to the urban context of Oyo and Ogun states [41,42]. Further, studies from Ogun and Oyo contexts demonstrate that targeted financial products (savings groups, rotating credit schemes) yield increased household resilience to shocks [43]. These financial tools would not only help residents improve their living standards, but also reduce their vulnerability to economic shocks and contribute to long-term community development.

### Climate resilience initiatives

Given the increasing climate risks faced by informal settlements, particularly extreme heat, flooding, and air pollution, community-based climate resilience initiatives are essential. These programmes should focus on sustainable land management, reforestation, water conservation, and disaster risk reduction [44]. By engaging communities in activities such as tree planting, waste recycling, and improving drainage systems, these initiatives can reduce the environmental degradation that often exacerbates living conditions in informal settlements [45].

Moreover, residents should be trained in climate-resilient agricultural practices to improve food security and reduce migration pressures caused by environmental degradation [46]. Climate-resilient housing, such as structures designed to withstand flooding or extreme heat, should also be prioritised. Governments can partner with international development organisations to provide technical expertise and funding for these programmes, ensuring that communities are better equipped to adapt to the impacts of climate change.

### Holistic and multi-sectoral approaches

Addressing the challenges in informal settlements requires a whole-of-society approach that integrates various sectors, including housing, health, education, and environmental sustainability. Policymakers, urban planners, NGOs, and private sector partners need to work together to design multi-component interventions that address the interconnected issues facing residents. For instance, slum upgrading should not only focus on physical infrastructure, but also include social programmes such as access to health care, education, and employment opportunities to ensure long-term improvement in the quality of life [47].

Additionally, fostering partnerships with academic institutions and research organisations can help generate more reliable data to inform policy decisions and monitor the impact of these interventions over time. Continuous evaluation and adaptation of programmes are necessary to ensure that they remain responsive to the evolving needs of informal settlement communities.

## CONCLUSIONS

The informal settlements in Ogun and Oyo states face complex and multifaceted health challenges driven by poor infrastructure, several environmental issues, and climate risks. The burden from respiratory illnesses, waterborne diseases, and heat-related conditions, exacerbated by inadequate health care access and substandard living conditions, underscores the urgent need for comprehensive interventions. Addressing these challenges requires an integrated and whole-of-society approach, combining improved infrastructure, enhanced public health interventions, and policies that mitigate climate-related risks, coupled with effective community participation. Lessons can be drawn from other African contexts, where targeted interventions in slum upgrading, climate adaptation, and health programmes have demonstrated success in reducing health risks. While various programmes and policies have been initiated in Oyo and Ogun states, significant barriers remain, including funding limitations, weak legislative frameworks, and the availability of reliable data. Overcoming these challenges will require a collaborative approach involving government agencies, NGOs, private sector partners, and communities. Engaging residents in urban planning and decision-making, promoting sustainable practices, and fostering strong stakeholder coordination will be essential for creating a more equitable and resilient urban environment.
